# La Real Expedicción Filantrópica de la Vacuna: entrevista con Susana Ramírez Martín

**DOI:** 10.1590/S0104-59702026000100025

**Published:** 2026-08-07

**Authors:** Susana María Ramírez Martín, Jaqueline Hasan Brizola, Tânia Salgado Pimenta, Gutiele Gonçalves dos Santos

**Affiliations:** i Profesora, Facultad de Ciencias de la Documentación/Universidad Complutense de Madrid. Madrid – España. sm.ramirez@pdi.ucm.es; ii Investigadora postdoctoral, Casa de Oswaldo Cruz/Fiocruz. Rio de Janeiro – RJ – Brasil. brizajaque@gmail.com; iii Investigadora, Casa de Oswaldo Cruz/Fiocruz. Rio de Janeiro – RJ – Brasil. tania.pimenta@fiocruz.br; iv Doctoranda, Casa de Oswaldo Cruz/Fiocruz. Rio de Janeiro – RJ – Brasil. gutielegoncalves12@gmail.com

**Keywords:** Susana María Ramírez Martín, Investigación, Historia de la ciencia, Salud pública, Francisco Xavier Balmis (1753-1719, Susana María Ramírez Martín, Research, History of science, Public health, Francisco Xavier Balmis (1753-1719

## Abstract

En esta entrevista, la profesora Susana Ramírez Martín habla de su trayectoria académica, relacionándola con los retos que supone dedicarse a un tema interdisciplinar, especialmente en España, donde la historiografía de la medicina y la salud se han desarrollado con una importante participación de médicos. También profundiza temas relacionados con su investigación sobre la Real Expedición Filantrópica de la Vacuna, aportando reflexiones comparativas sobre la introducción de la vacuna de Jenner en la América española y en la América portuguesa.

## Abstract (en)

In this interview, Professor Susana Ramírez Martín discusses her academic career and the challenges of pursuing an interdisciplinary field, especially in Spain. She highlights how the historiography of medicine and health has developed with significant participation from medical practitioners. She also explores topics from her research on the Royal Philanthropic Vaccine Expedition, offering comparative reflections on the introduction of Jenner’s vaccine in Spanish and Portuguese America.

Susana María Ramírez Martín es profesora titular del Departamento de Historia de América, Ciencias, Técnicas Historiográficas e Historia Medieval de la Facultad de Ciencias Documentales de la Universidad Complutense de Madrid. Es doctora por la Universidad Complutense de Madrid por su tesis *La Real Expedición Filantrópica de la Vacuna en la Real Audiencia de Quito*, 1999, dirigida por el profesor doctor José Luis Peset Reig. En 2016 realizó otro doctorado, también en la Universidad Complutense de Madrid, con la tesis *Presencia de la poliomielitis en el periódico “Voluntad” de Gijón (1950-1970)* (Ramírez Martín, 2016). Es autora de varios capítulos de libros y artículos en revistas científicas de alto impacto. Su primer libro publicado, *La salud del Imperio*: *la Real Expedición Filantrópica de la Vacuna* (Ramírez Martín, 2002), es una de las principales referencias en el continente europeo sobre la historia de la expedición filantrópica de la vacuna. La obra recibió su segunda edición en 2019. Sus intereses de investigación están relacionados principalmente con la historia de la medicina, la ciencia y la salud pública, así como con los archivos de las instituciones sanitarias como fuente de información y difusión del conocimiento científico entre los siglos XVIII y XIX.

*Profesora Susana, ¿cómo comenzó su interés por los temas relacionados con la historia de la medicina, en particular las vacunas y la salud pública en el contexto del Imperio español? ¿Podría hablarnos un poco de los principales hitos y retos que ha encontrado a lo largo de su carrera como investigadora?*

Mi primer trabajo fue ser maestra en un colegio de educación primaria. Estaba rodeada de niños de 9 a 10 años. En ese mismo momento, estaba estudiando la licenciatura en Historia de América, y, un día cualquiera, en un programa de televisión, escuché a Don Pedro Laín Entralgo hablar de los niños que participaron en la Real Expedición Filantrópica de la Vacuna. Mi primer pensamiento fue ver a mi clase metida en un barco durante las veinticuatro horas del día. Me estremecí. Ese sentimiento fue la chispa que motivó el querer saber más sobre este acontecimiento histórico.

Cuando me planteé los estudios de doctorado, lo tuve claro. Quería estudiar este hecho histórico y el contexto social, político, económico y cultural en el que se desarrolla. El primer hito que tuve que salvar fue encontrar director. Fue difícil encontrar un orientador para este tema mestizo, ya que necesitaba ser abordado desde la salud pública y desde la historia.

*En su autodescripción incluye la expresión «española de nacimiento, americanista de formación». ¿Cómo visualiza la historia de América en su formación? ¿Qué autores le han influido?*

Tuve la suerte de estudiar la licenciatura en la Universidad Complutense de Madrid, que, junto con la Universidad de Sevilla, era referente en los estudios de Historia de América dos años antes de la celebración del quinto centenario de su descubrimiento. Los primeros autores que influyeron en mi formación fueron mis profesores. Recuerdo con afecto a D. Mario Hernández Sánchez-Barba (América Contemporánea), D. José Manuel Pérez Prendes (Derecho Indiano), D. Alberto de la Hera (Historia de la Iglesia), D. Leoncio Cabrero (Descubrimientos geográficos), D. Jaime Delgado (América Moderna) y Dña. Concepción Bravo Guerreira (América Prehispánica), entre otros.

*¿Podría compartir con nosotros su visión de la historiografía latinoamericana dedicada a la historia de la salud y de la ciencia?*

Inicialmente, mi formación en historia de la salud y de la ciencia fue autodidacta. Empecé con un *Diccionario de terminología médica*, porque al principio no diferenciaba una pústula de una fístula o de un grano. Además, cerca de casa había una biblioteca que poseía un fondo bibliográfico especializado en temas americanistas. Era la Biblioteca del Instituto de Cooperación Iberoamericana, que en la actualidad es la Agencia Española de Cooperación Internacional y Desarrollo. En ella se reúne la mayor colección europea sobre tema americanista. En su maravillosa sala de investigadores pasé días interminables leyendo los libros de Aníbal Ruiz Moreno o de Gonzalo Díaz de Yraola, Francisco Fernández del Castillo, Ricardo Archila y los clásicos de Hipólito Unanue, José Felipe Flores, Andrés Bello y Saturnino Segurola.

*En las últimas décadas, varios investigadores españoles han realizado importantes aportaciones a la historia de la salud. Profesores como José María López Piñero y José Luis Peset Reig han abierto el camino a una generación de nuevos historiadores y médicos interesados en problematizar temas relacionados con las enfermedades, las epidemias y la salud pública, entre otros. ¿Qué importancia tienen las obras de Piñero y Reig para su trabajo?*

Tanto el profesor López Piñero como el profesor Peset Reig han sido ejemplos para mi investigación y referentes para los estudiosos de historia de la ciencia. El segundo fue mi director de tesis. A él le debo todo lo aprendido sobre el tema y una frase que ha sentenciado algunas reflexiones en mi vida: “Susana, lo mejor es enemigo de lo bueno”. Pepe Peset ha sido modelo académico y personal, porque a la calidad de su investigación se unen cualidades como la acogida, la caridad, la comprensión y la compasión. Fue aliento en momentos de desánimo y temple en los de euforia. Aprovecho para darle las gracias. Mi ser como investigadora no sería igual sin él.

*La Real Expedición Filantrópica de la Vacuna se describe a menudo como uno de los primeros esfuerzos mundiales de salud pública en proponer la vacunación masiva. ¿Podría hablarnos de las rutas de la expedición? ¿Y cómo repercutió en las poblaciones locales del Imperio español?*

La Real Expedición Filantrópica de la Vacuna exige a la Corona española un esfuerzo económico y demuestra que España es pionera en la preocupación por la salud pública de sus ciudadanos. La expedición estaba pensada como una unidad y tenía como objetivo llegar a todos los territorios hispanos. Cuando el convoy humanitario llega a Caracas, también llegan las dramáticas noticias de que la epidemia de Bogotá no estaba remitiendo.^1^ En ese momento, el director, doctor Balmis, divide la expedición en dos partes. Una ruta tomará el rumbo de América Septentrional dirigida por Balmis. Después de propagar la vacuna por la isla de Cuba, la península de Yucatán y la capitanía de Guatemala, llegaron a territorio novohispano por el puerto de Veracruz. Cuando llegaron a la capital novohispana se percataron de que el fluido había llegado antes que ellos. La desilusión fue grande. Balmis y su grupo llevaron el fluido por el Camino Real de Tierra Adentro desde la Ciudad de México hasta la población de Durango, pasando por Puebla, Oaxaca, Querétaro, Guanajuato, Valladolid, Guadalajara y Zacatecas. Decidió no seguir la vacunación por la Comanchería por los constantes ataques entre los indios apaches y comanches. Ya de vuelta en la Ciudad de México, se dedicaron a preparar el viaje en el Galeón de Manila para cruzar el Pacífico con destino a la capital de Filipinas.^2^

La otra ruta tomará el rumbo a América Meridional. El camino era desconocido para todos los expedicionarios y no tuvieron buena suerte. El barco que les transportaba encalló en la ciudad de Barranquilla en la desembocadura del río Magdalena. Siguiendo el cauce del río llegaron a Bogotá. Tardaron más tiempo del previsto y a partir de ese momento hicieron la ruta hacia el sur por dos caminos paralelos, por la sierra y por la costa. El esfuerzo físico fue grande por la complejidad de la geografía a la que se tenían que enfrentar. Las cifras abruman: los seiscientos quilómetros de distancia que separan Santa Fe de Bogotá de Popayán y los 250km de Popayán a Pasto, que salvan un desnivel de dos mil metros. Pasaron a la Real Audiencia de Quito cruzando el Ecuador terrestre por Ipiales e Ibarra, sobrecogidos por las cimas nevadas de los volcanes Cayambe y Antisana. Llegaron extenuados y tuvieron que reponer fuerzas en la ladera del Pichincha. Poco duró el descanso. Después de ochocientos quilómetros escoltados por los volcanes Cotopaxi, Tungurahua y Chimborazo, la expedición llega a la ciudad de Piura en el Virreinato del Perú. La capital peruana estaba lejos, a más de mil quilómetros. Pasaron por las ciudades de Cajamarca, Trujillo y Chimbote. Llegaron a Lima consumidos por el esfuerzo realizado y con la salud desgastada. Desde Lima dos sanitarios toman el rumbo a la ciudad de Buenos Aires y los otros dos toman camino hacia la ciudad de Santiago en la Capitanía General de Chile.

Para la realización de esta hazaña se necesitó, además de la implicación del Consejo de Indias y mucho entusiasmo, la participación de las autoridades locales que apoyaron económicamente y favorecieron anímicamente el proyecto sanitario.^3^

*¿Podemos hablar de los retos a los que se enfrentó la Real Expedición, especialmente en lo que se refiere a la aceptación o resistencia a la vacuna?*

La docilidad o no en la aceptación de la vacuna variaba en función de la formación y la capacidad de persuasión de los médicos que tenían que administrarla y de los párrocos que tenían que predicarla. Podemos hablar de que el proceso de sensibilización tenía como destinatarias a las madres, que eran las responsables directas de la salud de las familias. Esto se pone de manifiesto explícitamente en la dedicatoria de la traducción que Balmis hace en 1803 del *Tratado histórico práctico de la vacuna*: *“a las madres de familia”*.^4^

*¿Cuál es la relevancia de la Real Expedición Filantrópica de la Vacuna para la historia global de la salud pública?*

La relevancia más significativa de la Real Expedición Filantrópica de la Vacuna es la científico-médica. Podemos afirmar que se establecen las bases de los protocolos de las campañas vacunales, que se han mantenido hasta el siglo XXI. Los efectos sanitarios rápidamente se manifestaron, ya que disminuyó la tasa de mortalidad y favoreció el crecimiento vegetativo de la población. Además de ese impacto inmediato, existe un legado científico porque define una urdimbre vacunal que implica a los facultativos locales formados en Ultramar. Se crean Juntas de Vacuna vinculadas a instituciones de prestigio como las universidades y las Reales Sociedades de Amigos del País, ya que ser miembro de ellas otorgaba prestigio social.^5^ Desde estas instituciones creadas para la perpetuación de la vacuna, se fomenta la correspondencia entre los diferentes territorios y se consolidan las rutas de comunicación, en las que se difunden no solo ideas científicas, sino también políticas y sociales.

*Sabemos que la “Real expedición de la Vacuna” no incluyó Brasil, ya que fue una iniciativa de la corona española. Sin embargo, responsables del Imperio portugués que actuaban en capitanías estratégicas en Brasil dejaron importantes informes sobre sus conocimientos de la expedición de Balmis y Salvany en los primeros años del siglo XIX. ¿Cómo evalúa la circulación del conocimiento científico relacionado con la vacuna en este período?*

El proyecto sanitario hispano no incluye el territorio brasileño porque en ese momento histórico las dos coronas eran enemigas. El 29 de enero de 1801 se firmó el Tratado de Madrid entre España y Francia para obligar a Portugal a abandonar su alianza con Inglaterra o invadirla si no lo hiciera. Portugal no aceptó las condiciones y el 27 de febrero España declaró la guerra a Portugal. Tres meses más tarde, el 20 de mayo, el Ejército español invadió el territorio portugués, dando comienzo a la efímera Guerra de las Naranjas. El 6 junio de ese mismo año, Portugal firmó con España y Francia el tratado de Badajoz, por el que se acordaba el fin de la guerra. Esta inseguridad diplomática como consecuencia de estos vaivenes internacionales provocó la necesidad de salvoconductos para que la Real Expedición Filantrópica de la Vacuna navegase por el Atlántico con seguridad. El conflicto europeo no se percibía de igual manera en Ultramar, donde el movimiento de mercancías entre territorios hispanos y lusos era fluido. La vacuna se intercambió como una mercancía más. La vacuna llegó al puerto de Salvador de Bahía en territorio brasileño el 30 de diciembre de 1803 por iniciativa de Felisberto Caldeira Brant Pontes de Oliveira (1772-1842).^6^ Desde allí se propagó rumbo al sur por la costa. Desde Río de Janeiro llegó la vacuna a Buenos Aires en brazos de esclavos negros que estaban a bordo de la fragata La Rosa del Río, capitaneada por Manuel Joseph Díaz. La travesía hasta el río de la Plata duró poco más de 15 días, se inició el 17 de junio de 1804 y terminó el 5 de julio del mismo año. Las noticias se publicaron en el periódico bonaerense titulado *Semanario de Agricultura, Industria y Comercio*.^7^

*A partir de su investigación, ¿es posible identificar una causa para la falta de diálogo entre los Reinos de España y Portugal en lo que se refiere a la no inclusión de Brasil en la ruta de la expedición?*

En un contexto inmediato, la primera explicación es el posicionamiento de cada país con relación al enfrentamiento bélico franco-británico. Portugal era aliado de Inglaterra, y España era enemiga. Si profundizamos en la reflexión y tomamos como referencia un periodo histórico más amplio, podemos afirmar que las rutas de navegación no relacionaban los territorios hispanos y lusos desde la conquista hasta la entrada en funcionamiento del Reglamento de Libre Comercio en 1778.^8^ A partir de este momento, los puertos de Buenos Aires y Montevideo en el río de la Plata se abren al comercio internacional, pero sus rutas son débiles.

*En la introducción de su libro La salud del Imperio usted afirma que la introducción de la vacuna cambió la mentalidad científica colonial. Sin embargo, algunos estudios demuestran la coexistencia de la vacuna animal con antiguos métodos de tratamiento contra la viruela, como el variolización, que ha avanzado mucho en países como Brasil, precisamente porque se practicaba desde mucho tiempo y porque dialogaba con la cultura de los pueblos africanos e indígenas. ¿Cómo ve esta cuestión?*

Pienso que el descubrimiento de la vacuna no supone un rechazo de otras técnicas utilizadas hasta el momento. La inoculación de la viruela vacuna convive con la inoculación de la viruela humana o variolización. Además, en América se usan procedimientos medievales para aislar poblaciones: los cordones sanitarios y las cuarentenas. Al mismo tiempo, para mitigar los efectos de la viruela se administran remedios (pomadas y ungüentos) heredados de la medicina indígena.

*Sobre la documentación relativa a la historia de la expedición de la vacuna en España, ¿cuáles serían los principales archivos a consultar?*

La producción documental generada por la Real Expedición Filantrópica de la Vacuna se desarrolla en dos contextos. El primero es el estatal, conservado en la Península, que reúne los documentos producidos y recibidos por el Consejo de Indias en el ejercicio de sus funciones. El expediente sobre la introducción de la vacuna en América se custodia en el legajo 1558A de la sección Indiferente General del Archivo General de Indias, que está en Sevilla (España). El segundo es el local, que reúne los documentos relacionados con la propagación y perpetuación de la vacuna en cada uno de los territorios y localidades por las que pasa la expedición. En el archivo municipal de todas y cada una de las ciudades recorridas por los expedicionarios se conservan documentos. Listas de vacunaciones realizadas, libros de actas de las diferentes Juntas de Vacuna creadas o estadillos e informes ofrecen una información específica de cada población.

*¿Cómo el estudio de las campañas de vacunación en la época colonial nos permite comprender los retos a los que nos enfrentamos en las campañas de vacunación actuales, como las relacionadas con covid-19? ¿Puede, la investigación del pasado, ayudarnos a pensar en el presente?*

La Real Expedición Filantrópica de la Vacuna es la primera campaña de vacunación organizada. La labor realizada no se limitó al mero transporte del fluido vacuno por los territorios, sino que diseñó un sistema organizativo que tuvo éxito y sirvió de modelo para la perpetuación de la vacuna en diferentes territorios.^9^ Los protocolos de actuación constituyen una estrategia que será replicada durante 200 años y guarda similitud con los modernos modelos de planificación sanitaria de Raynald Pineault y Carole Daveluy, publicados en 1986. En cualquier planificación sanitaria participa un avance científico y necesita un equilibrio entre el conocimiento adquirido y la documentación generada. El programa de salud diseñado por Balmis es arquetípico porque tiene los objetivos claros, posibles y verificables, presenta un inventario de actividades secuenciadas y jerarquizadas para evidenciarlos y posee los recursos económicos, políticos y sociales necesarios para llevar a cabo el programa.

*Ante el rechazo de los potenciales beneficios de las vacunas en diversos países del mundo en la actualidad, y teniendo en cuenta el comportamiento de las personas que vivieron en el siglo XIX, ¿diría que se ha producido un retroceso en términos de vulgarización científica? ¿Pueden compararse las razones que llevaron a la gente en el pasado a rechazar la vacuna con las cuestiones contemporáneas? ¿Es posible establecer un diálogo entre las épocas?*

En el paso del siglo XVIII al XIX, la desconfianza, el escepticismo y la ignorancia, primero frenan y después retardan la difusión de la vacuna. Desde que Jenner publicó su descubrimiento para erradicar las viruelas, la vacuna contó con partidarios y con detractores. Moreau de la Sarthe en su libro titulado *Tratado histórico práctico de la vacuna*, traducido por Balmis al español en 1803, poco antes del inicio de la Real Expedición Filantrópica de la Vacuna, enumera cuatro tipos de actitudes: la de los ignorantes que “se burlaron”, la de los entusiastas que “lo aprobaron sin examen, la de los sabios que “se resistieron a creerlo” y la de los prudentes que “pidieron tiempo y nuevos experimentos”. Creo que estos patrones que se definieron en aquel momento se mantienen hoy en día. Estas actitudes son transversales a profesiones, ideales políticos, capacidad económica o formación académica.

*Recientemente, los debates en torno a la salud pública han animado a los historiadores a dar a conocer sus investigaciones más allá de los círculos académicos. ¿Cree en la función social de la historia de la salud? ¿Qué barreras debemos derribar para establecer un diálogo con un público más amplio?*

Considero que la investigación sin difusión no tiene sentido, porque los nuevos aprendizajes no revierten en la sociedad ni la conforman. Al conocer la historia de la salud pública, se visibilizan los hechos históricos y se democratiza el conocimiento. Las actividades de difusión que se realicen tienen que estar planificadas y pensadas para todos los públicos. La difusión no debería partir exclusivamente del ámbito universitario, sino que debería dialogar e interactuar con otras instituciones, tanto públicas como privadas. Yo creo que cuando difundimos, deberíamos acercar los frutos de la investigación tanto al experto como al profano, con el fin de fomentar inquietudes ofreciendo alternativas de formación y entretenimiento.

## Data Availability

No están en repositorio.

## NOTAS

1 Sobre la llegada de la vacuna en Colombia véase Ramirez Martín (2007).

2 Véase Ramirez Martín (2004a) y Mark y Rigau-Pérez (2009, p.67).

3 Para más informaciones sobre cómo se recibió la vacuna en los distintos lugares mencionados por la entrevistada, véase: Ramírez Martín y Ballester Añón (2010, p.133-135); Thompson (1993); García Nieto y Hernández (2005); Colvin (2006); Denis (2007). Los detalles de las rutas alcanzadas por Balmis y Salvany pueden consultarse en Ramirez Martín (2010); Pascual (2010); y Mark y Rigau-Pérez (2009, p.67).

4 Tratado histórico y práctico de la vacuna es un libro escrito por Jacques Louis Moreau de la Sarthe (1771-1826) y traducido por Francisco Xavier de Balmis. El libro recoge los resultados de observaciones y experimentos sobre la vacuna, además de analizar sus ventajas y las objeciones a la eficacia del método.

5 Sobre las juntas de vacuna y sus funciones tras la llegada de la expedición de Balmis, consultar Ramirez Martín (2004b, p.52).

6 Sobre la llegada de la vacuna en ciudades brasileñas en principios del siglo XIX véase Portugal (2018), Hochman y Souza (2022) y Brizola (2024).

7 Este periódico se publicó semanalmente entre 1802 y 1807 en Buenos Aires, entonces capital del Virreinato del Río de la Plata.

8 En 1778 se produjo un importante cambio en la organización del comercio colonial español con América. Los decretos de libre comercio pusieron fin al sistema de puerto único vigente desde principios del siglo XVI, capitalizado por Sevilla y Cádiz. A partir de entonces, una serie de puertos españoles pudieron comerciar directamente con América. Los detalles de este acontecimiento histórico pueden consultarse en Iglesias Rodríguez (2011).

9 Consultar el libro organizado por la entrevistada durante la pandemia de covid-19 (Ramirez Martín, 2021).

## References

[B1] BRIZOLA Jaqueline (2024). Entre bozales e sujeitos de cor: a vacinação contra a varíola nas cidades do Rio de Janeiro e La Habana (1804-1808). Anais do Instituto de Higiene e Medicina Tropical.

[B2] COLVIN Thomas (2006). Arms around the world. The introduction of smallpox vaccine into the Philippines and Macao in 1805. Revista de Cultura.

[B3] DENIS Adrián López (2007). Disease and society in colonial Cuba, 1790-1840.

[B4] GARCÍA NIETO Víctor, HERNÁNDEZ Justo (2005). La Real Expedición Filantrópica de la Vacuna en Canarias (9 de diciembre de 1803 6 de enero de 1804). Asclepio.

[B5] HOCHMAN Gilberto, SOUZA Christiane Maria Cruz de (2022). Vacina e Vacinação antivariólica na Bahia Oitocentista. Ciência & Saúde Coletiva.

[B6] IGLESIAS RODRÍGUEZ Juan José, Sánchez Mantero R., Erausquin Estela (2011). España y América en el bicentenario de la Independencia: miradas sobre lo extraño y el extranjero.

[B7] MARK Catherine, RIGAU-PÉREZ José (2009). The world's first immunization campaign: the Spanish smallpox vaccine expedition, 1803-1813. Bulletin of the History of Medicine.

[B8] MOREAU Jacques Louis, Balmis Dr. Francisco Xavier de (1803). Tratado histo´rico y pra´ctico de la vacuna: que contiene en compendio el origen y los resultados de las observaciones y experimentos sobre la vacuna con todo lo demás que concierne a la práctica del nuevo modo de inocular.

[B9] PASCUAL Emilio Soler (2010). Canelobre: Revista del Instituto Alicantino de Cultura "Juan Gil-Albert".

[B10] PORTUGAL Fillipe dos Santos (2018). A institucionalização da vacina antivariólica no Império Luso-brasileiro nas primeiras décadas do Século XIX.

[B11] RAMÍREZ MARTÍN Susana (2021). La expedición de Balmis: primer modelo de lucha global contra las pandemias.

[B12] RAMÍREZ MARTÍN Susana (2016). Presencia de la poliomielitis en el periódico "Voluntad" de Gijón: (1950-1970).

[B13] RAMÍREZ MARTÍN Susana (2010). Desarrollo geográfico de la Real Expedición Filantrópica de la Vacuna (1803-1821). Canelobre: Revista del Instituto Alicantino de Cultura "Juan Gil-Albert".

[B14] RAMÍREZ MARTÍN Susana (2007). Propagación y perpetuación de la vacuna contra la viruela en Nueva Granada. Boletín de Historia y Antigüedades de Colombia.

[B15] RAMÍREZ MARTÍN Susana (2004a). La Real Expedicio´n Filantro´pica de la Vacuna: doscientos an~os de lucha contra la viruela.

[B16] RAMÍREZ MARTÍN Susana (2004b). El legado de la Real Expedición Filantrópica de la Vacuna (1803-1810): las juntas de vacuna. Asclepio: Revista de Historia de la Medicina y de la Ciencia.

[B17] RAMÍREZ MARTÍN Susana (2002). La salud del Imperio: la Real Expedición Filantrópica de la Vacuna.

[B18] RAMÍREZ MARTÍN Susana María, BALLESTER AÑÓN Rosa (2010). Los miedos a la viruela y sus comportamientos sociales en la metrópoli y en las colonias americanas. Canelobre: Revista del Instituto Alicantino de Cultura "Juan Gil-Albert".

[B19] THOMPSON Angela (1993). To save the children: smallpox inoculation, vaccination, and public health in Guanajuato, Mexico, 1797-1840. The Americas.

